# Objectively measured physical activity and kidney function in older men; a cross-sectional population-based study

**DOI:** 10.1093/ageing/afx091

**Published:** 2017-06-01

**Authors:** Tessa J Parsons, Claudio Sartini, Sarah Ash, Lucy T Lennon, S Goya Wannamethee, I-Min Lee, Peter H Whincup, Barbara J Jefferis

**Affiliations:** 1 UCL Department of Primary Care and Population Health, UCL Medical School, London NW3 2PF, UK; 2 Brigham and Women's Hospital, Harvard Medical School, Boston, MA 02215, USA; 3 Population Health Research Institute, St George's University of London, Cranmer Terrace, London SW17 0RE, UK

**Keywords:** older people, physical activity, kidney function, sedentary behaviour, glomerular filtration rate

## Abstract

**Background:**

kidney function declines in older adults and physical activity levels are low. We investigated whether higher levels of physical activity and lower levels of sedentary behaviour were associated with lower odds of low kidney function in older men.

**Methods:**

cross-sectional study of 1,352 men from the British Regional Heart Study, mean (standard deviation) age 78.5 (4.6) year. Physical activity and sedentary behaviour were measured using Actigraph GT3X accelerometers. Kidney function was measured by estimated Glomerular filtration rate (eGFR) using the chronic kidney disease-EPI creatinine-cystatin equation. Associations between physical (in)activity and kidney function were investigated using regression models.

**Results:**

higher levels of physical activity and lower levels of sedentary behaviour were associated with reduced odds ratios (ORs) for lower eGFR (<45 versus ≥45 ml/min per 1.73 m^2^) after adjustment for covariates. Each additional 1,000 steps, 30 min of light physical activity and 10 min of moderate/vigorous physical activity per day were associated with a lower odds (95% confidence interval (CI)) of a low eGFR; OR 0.81 (0.73, 0.91), OR 0.87 (0.78, 0.97) and OR 0.84 (0.76, 0.92), respectively. Each additional 30 min of sedentary behaviour per day was associated with a higher odds of a low eGFR (1.16 95% CI 1.06, 1.27). Associations between moderate/vigorous physical activity and lower kidney function persisted after adjustment for light physical activity or sedentary behaviour.

**Conclusion:**

physical activity is associated with kidney function in older men and could be of public health importance in this group who are at increased risk of poor kidney function and low physical activity. More evidence is needed on whether the association is causal.

## Introduction

Kidney function declines with age and predicts adverse outcomes such as mortality and cardiovascular disease [1, 2]. Kidney function and chronic kidney disease (CKD) are commonly defined by glomerular filtration rate (GFR), usually estimated by one of several equations based on age, gender, race and serum creatinine and/or cystatin C.

The most common causes of kidney failure (the most severe stage of CKD), are diabetes and hypertension [3]. Modifiable factors that influence kidney function even at low levels could be of public health importance, especially for older age groups. Physical activity is known to be protective against CKD risk factors such as diabetes, hypertension and cardiovascular disease [4] and exercise is recommended in management strategies for people diagnosed with CKD [5, 6, 7]. Observational studies have predominantly used self-report measures of physical activity, which are limited in a number of respects and tend to be less reliable in older adults [8]. Mixed findings have been reported across cross-sectional and longitudinal designs, with some studies reporting associations [9, 10] and others not [11, 12], although the direction of the relationships is consistent (physical activity associated with a lower risk of CKD). However, there is a paucity of studies with objectively measured (in)activity.

We investigated associations between objectively measured (in)activity, sedentary behaviour and kidney function, as measured by estimated (e)GFR, using a community sample of older British men.

## Research Design and Methods

### Sample

The British Regional Heart Study is a population-based cohort study following up 7,735 men recruited from primary care practices in 1978–80. Between 2010 and 2012, all 3,137 survivors were invited to a physical examination, to provide a blood sample, complete a general questionnaire and wear a physical activity monitor (accelerometer), as described more fully elsewhere [13]. The National Research Ethics Service Committee London provided ethical approval. Participants provided informed written consent to the investigation in accordance with the Declaration of Helsinki.

### Measures

GFR, expressed as ml/min per 1.73 m^2^ was estimated from serum creatinine and cystatin C using the CKD Epidemiology Collaboration Creatinine-Cystatin (CKD-EPI cr-cys) equation [14]. Almost all the men (>99%) were White European.

Men wore the GT3X accelerometer (Actigraph, Pensacola, Florida) over the right hip for 7 days, during waking hours, removing it for swimming or bathing. Men with ≥3 days of ≥600 min wear time (an accepted standard for compliant wear) were included in analyses. Each minute of activity was categorised using intensity threshold values of counts per minute developed for older adults: <100 for sedentary behaviour (<1.5 metabolic equivalent (MET)), 100–1,040 for light activity (1.5–3 MET) and >1,040 for moderate/vigorous activity (≥3 MET) [15].

Body mass index (BMI, kg/m^2^) and systolic blood pressure (SBP) were measured. Men completed a questionnaire on current lifestyle factors and medication use, and recorded whether they had ever received a doctor diagnosis of heart attack, heart failure or stroke. Social class was based on longest held occupation at study entry and categorised as manual and non-manual [16].

### Statistical methods

Men with kidney failure, defined by estimated GFR (eGFR) < 15 ml/min per 1.73 m^2^ were excluded from analyses (*n* = 2). Descriptive statistics for demographic characteristics, physical activity and sedentary behaviour, were calculated by category of kidney function (based on eGFR). We investigated associations using logistic regression models for lower kidney function using a cut-off suggested for older age groups (eGFR < 45 versus eGFR ≥ 45 ml/min per 1.73 m^2^) [17]. We examined each physical activity exposure separately: total activity counts per day, steps per day and minutes per day of sedentary behaviour, light activity and moderate/vigorous activity, and then (i) moderate/vigorous activity and sedentary behaviour and (ii) moderate/vigorous activity and light activity in the same model. Sedentary behaviour and light activity were not included in the same model due to collinearity (*r* = −0.62). We estimated odds ratios (ORs) for each 10,000 counts of total activity, 1,000 steps, 30 min of sedentary behaviour or light activity and 10 min of moderate/vigorous activity.

Models were adjusted for average accelerometer wear time (minutes/day), age, age squared (Model 1) plus season of accelerometer wear (warm, May–September or cold, October–April), region of residence, social class, living alone, smoking status, alcohol consumption, use of statins and anti-hypertensives, existing cardiovascular disease, diabetes, SBP and total cholesterol (Model 2) plus BMI and C-reactive protein (CRP) (Model 3).

## Results

Of 3,137 men invited to the physical examination, 1,722 (55%) attended, 1,424 had both eGFR and (in)activity measures of whom 2 were excluded due to eGFR < 15 ml/min per 1.73 m^2^. Our main analyses included 1,350 men with complete data on covariates. Mean (standard deviation) eGFR among all men was 72.0 (22.1) ml/min per 1.73 m^2^. Of men who were invited to the examination, those with complete data and included in our study (*n* = 1,350) had a lower BMI 10 years earlier, 26.7 versus 27.3 kg/m^2^, and were more active, 59% versus 46% at least moderately active, than those who did not attend or did not have complete data (*n* = 1,787 for BMI, *n* = 1,293 for physical activity questionnaire).

Men with lower eGFR spent more time in sedentary behaviour and less time in physical activity (Table 1). These men were older, and scored less favourably on a number of lifestyle factors and medication use (Table 1).

**Table 1.** Characteristics of 1,419 men by category of kidney function (eGFR using CKD-EPI creatinine-cystatin equation). Figures are mean (standard devation) unless stated otherwise

		eGFR (ml/min per 1.73 m^2^)			*P* (trend)	All men	*N*
	15 to <45 (*n* = 148)^a^	45 to <60 (*n* = 258)^a^	60 to <90 (*n* = 759)^a^	≥90 (*n* = 254)^a^			
Age (years)	81.8 (5.4)	80.5 (4.7)	78.0 (4.1)	76.1 (3.5)	<0.0001	78.5 (4.6)	1,419
Manual social class,% (*n*)	54 (80)	52 (132)	45 (338)	44 (110)	0.05	47 (676)	1,406
Lives alone, % (*n*)	29 (42)	21 (52)	20 (148)	13 (33)	0.002	20 (280)	1,399
Smoker (current/recent), % (*n*)	7.6 (11)	9.7 (25)	7.7 (58)	5.5 (14)		7.7 (108)	
Long term ex smoker, % (*n*)	59.3 (86)	58.8 (151)	52.8 (398)	50.0 (127)	0.05	54 (762)	1,410
Taking statins, % (*n*)	55 (81)	52 (135)	48 (365)	53 (134)	0.3	50 (730)	1,419
Taking anti-hypertensives % (*n*)	76 (113)	66 (171)	54 (408)	52 (131)	<0.0001	58 (849)	1,419
Cardiovascular disease % (*n*)	28 (41)	19 (49)	14 (110)	14 (36)	<0.0001	17 (244)	1,419
Diabetic % (*n*)	26 (39)	17 (44)	11 (87)	18 (45)	<0.0001	15 (219)	1,419
Alcohol (units per week)	5.5 (7.7)	4.6 (6.3)	6.3 (7.5)	7.9 (8.4)	<0.0001	6.2 (7.5)	1,385
BMI (kg/m^2^)	27.5 (4.6)	27.2 (3.6)	26.9 (3.6)	27.0 (4.1)	0.1	27.1 (3.8)	1,406
SBP (mm Hg)	141 (22)	146 (19)	147 (19)	149 (18)	<0.0001	146.5 (19.1)	1,416
Total cholesterol (mmol/l)	4.2 (1.0)	4.4 (1.0)	4.7 (1.0)	4.8 (1.0)	<0.0001	4.6 (1.0)	1,419
CRP (mg/l)^b^	2.95 (3.29)	1.70 (3.40)	1.20 (3.03)	0.89 (3.40)	<0.0001	0.29 (1.21)	1,416
Cystatin (mg/l)^b^	1.73 (1.21)	1.28 (1.12)	0.97 (1.15)	0.57 (1.55)	<0.0001	0.99 (1.48)	1,419
Creatinine (μmol/l)^b^	150.4 (1.2)	110.1 (1.1)	88.9 (1.1)	77.8 (1.1)	<0.0001	95.63 (1.27)	1,419
Accelerometer wear time (min/day)	834 (70)	837 (67)	860 (64)	858 (71)	<0.0001	853 (68)	1,419
Total activity (counts per day)	100,296 (69,394)	128,316 (80,728)	173,534 (97,799)	196,031 (111,197)	<0.0001	160,702 (99,019)	1,419
Steps/day	3,073 (2,109)	3,882 (2,327)	5,215 (2,770)	5,812 (3,040)	<0.0001	4,831 (2,807)	1,419
% time spent sedentary	78 (9)	75 (9)	71 (9)	70 (9)	<0.0001	73 (9)	1,419
% time in light activity	19 (7)	22 (7)	24 (7)	25 (7)	<0.0001	23 (7)	1,419
% time in moderate/vigorous activity	2.4 (2.5)	3.5 (3.2)	4.9 (3.7)	5.8 (4.2)	<0.0001	4.5 (3.7)	1,419
Sedentary behaviour (min/day)	652 (77)	625 (84)	613 (82)	597 (81)	<0.0001	617 (84)	1,419
Light activity (min/day)	161 (63)	182 (63)	204 (63)	211 (68)	<0.0001	196 (66)	1,419
Moderate/vigorous activity (min/day)	21 (22)	30 (28)	43 (32)	50 (37)	<0.0001	39 (33)	1,419

Pearson chi square test used for all categorical variables except smoking for which Fisher's exact test was used.

^a^Maximum *N* in quartile, varies slightly with missing covariate data.

^b^Geometric means given.

In regression models, higher levels of total counts, steps, moderate/vigorous activity or light activity were associated with a reduced odds, and higher levels of sedentary behaviour with a higher odds of low eGFR (<45) (Table 2, Model 1). Associations were only slightly attenuated after adjusting for all covariates (Model 2). For example, the adjusted odds of low kidney function was 0.84 (95% confidence interval (CI) 0.76, 0.92) for an additional 10 min of moderate/vigorous activity per day. An additional 30 min of sedentary behaviour per day was associated with an increased odds for low eGFR of 1.16 (95% CI 1.06, 1.27). In models including moderate/vigorous activity and sedentary behaviour or moderate/vigorous activity and light activity simultaneously, associations with low eGFR were slightly attenuated but remained significant for moderate/vigorous activity (Table 2). Adjusting models for BMI and CRP slightly further attenuated associations (Table 2, Model 3). Repeating models using alternative equations to calculate eGFR resulted in broadly similar findings, although with the CKD-EPI cystatin equation sedentary behaviour was associated with eGFR independently of moderate/vigorous activity rather than vice versa (see Supplementary data, Table Appendix 1, available at *Age and Ageing* online).

**Table 2.** Cross-sectional associations between physical activity intensity, sedentary time and eGFR (<45 versus ≥45 ml/min per 1.73 m^2^) using CKD-EPI creatinine-cystatin equation

	Model 1	*n* = 1,419	Model 2	*n* = 1,350	Model 3	*n* = 1,335
	OR	(95% CI)	OR	(95% CI)	OR	(95% CI)
Total vertical counts(per 10,000/day)	**0.92**	**(0.89, 0.95)**	**0.93**	**(0.90, 0.97)**	**0.95**	**(0.92, 0.98)**
Steps (per 1,000/day)	**0.76**	**(0.69, 0.84)**	**0.81**	**(0.73, 0.91)**	**0.85**	**(0.77, 0.95)**
Moderate/vigorous activity (10 min/day)	**0.80**	**(0.72, 0.88)**	**0.84**	**(0.76, 0.92)**	**0.87**	**(0.78, 0.96)**
Light activity (30 min/day)	**0.81**	**(0.73, 0.89)**	**0.87**	**(0.78, 0.97)**	**0.90**	**(0.81, 1.00)**
Sedentary behaviour (30 min/day)	**1.23**	**(1.13, 1.33)**	**1.16**	**(1.06, 1.27)**	**1.12**	**(1.03, 1.23)**
Moderate/vigorous activity (10 min/day)	**0.87**	**(0.77, 0.98)**	**0.88**	**(0.77, 1.00)**	0.90	(0.79, 1.02)
Sedentary behaviour (30 min/day)	1.13	(1.01, 1.25)	1.07	(0.95, 1.20)	1.05	(0.93, 1.18)
Moderate/vigorous activity (10 min/day)	**0.84**	**(0.75, 0.92)**	**0.86**	**(0.77, 0.96)**	**0.88**	**(0.79, 0.98)**
Light activity (30 min/day)	**0.89**	**(0.80, 0.99)**	0.94	(0.83, 1.05)	0.95	(0.85, 1.07)

Bold type indicates significance at the 5% level. Model 1 adjusted for daily accelerometer wear time, age and age squared. Model 2 adjusted as for Model 1 plus season of wear, region of residence, social class, living alone, tobacco, alcohol consumption, statin use, antihypertensive use, cardiovascular disease, diabetes, SBP and total cholesterol. Model 3 adjusted as for Model 2 plus BMI and CRP.

## Discussion

In our study of older community dwelling men, all the (in)activity measures we investigated were associated with kidney function after adjustment for covariates; higher levels of physical activity and lower levels of sedentary behaviour were associated with a lower odds of eGFR < 45 ml/min per 1.73 m^2^. These findings are clinically relevant since physical activity and sedentary behaviour are modifiable, and may be particularly important for older people, in whom kidney function is declining [18, 19].

Higher levels of moderate/vigorous activity were associated with eGFR independently of sedentary behaviour or light activity, although the association between moderate/vigorous activity and eGFR was not independent of sedentary behaviour after additional adjustment for BMI and CRP. Very few studies have examined objectively measured (in)activity in relation to kidney function [9, 20]. In the NHANES study, in men without diabetes, moderate/vigorous activity, light activity and sedentary behaviour were each associated with kidney function, moderate/vigorous activity and light activity and moderate/vigorous activity and sedentary behaviour independently of each other, but these associations were not present in men with diabetes [20]. A longitudinal study of adults with recently diagnosed Type 2 diabetes also found moderate/vigorous activity and sedentary behaviour were associated with decreased and increased risk respectively of developing CKD, but these associations were not independent [9].

A major strength of our study is the use of accelerometers to investigate physical activity of different intensities in relation to kidney function. We use cystatin C and creatinine to estimate eGFR; while creatinine levels are widely used to estimate kidney function, cystatin C is less influenced by muscle mass and dietary intake. Our population of older men is community-based which increases the generalisability of our results beyond clinical groups, although possibly not to women or younger age groups, since kidney function levels and associations between (in)activity and kidney function may differ by gender and age [10, 20, 21]. We were able to account for a range of potential confounders, including BMI and CRP. Poorer kidney function is associated with increased inflammation [22] and increased BMI (BMI particularly in older age groups) [23]. We found associations between (in)activity to be reduced but not eliminated after adjusting for BMI and/or CRP, suggesting that increased inflammation and/or BMI may be partial mediators. Our data are limited in being cross-sectional, and since kidney disease can cause fatigue and weakness, associations may operate in either or both directions; lower kidney function may lead to less physical activity and more sedentary behaviour, which may explain some of the inconsistencies seen across studies.

This study found higher levels of physical activity and lower levels of sedentary behaviour were associated with eGFR < 45 ml/min per 1.73 m^2^ in older men. Since older age groups are at higher risk of poor kidney function due to the age-related decline in kidney function, and because they also have low physical activity levels, they may have greater potential to benefit from increasing their levels of physical activity or reducing levels of sedentary behaviour.

**Key points**

- Kidney function declines in older adults and physical activity levels are low.
- Higher levels of physical activity and lower levels of sedentary behaviour were beneficially associated with kidney function.
- Moderate/vigorous activity was beneficially associated with kidney function after adjusting for light activity or sedentary behaviour.

## Supplementary Material

Supplementary Data
