# Supplementary material for: Objectively measured physical activity and kidney function in older men; a cross-sectional population-based study
Source: Age Ageing. 2017 Jun 1;46(6):1010–4. doi: 10.1093/ageing/afx091 (PMC5860422; doi:10.1093/ageing/afx091)
Supplement: Supplementary Data [file afx091_aa-17-0045-file001.docx]

**Supplementary Data**

**Methods**

Fasting venous blood samples were collected. Creatinine was measured using a rate-blanked kinetic Jaffé assay, with a coefficient of variation (CV) of 2.3%. Cystatin C was measured on a SPAPLUS®automated analyzer (The Binding Site Group, Ltd), with a limit of detection of 0.4 mg/L and CV range of 5.4–9.4%. Men with cystatin C below the limit of detection were assigned a value of 0.2 mg/L (50% of the functional limit of detection). C-reactive protein (CRP, mg/l) was assayed using ultrasensitive assay (e411, Roche, Burgess Hill, UK) using the manufacturers calibrators and controls (CV 6.9%). The distributions of creatinine, cystatin C and CRP were skewed and therefore transformed using natural logarithm for the purpose of descriptive statistics (Table 1).

Accelerometer data were processed using standard methods.^1^ Non-wear time was excluded using the R package “Physical Activity”.^2^

Body mass index (BMI, kg/m^2^) was calculated from height (Harpenden stadiometer) and weight in light indoor clothing (Tanita body composition analyser (BC-418) or Tanita scales if the participant had a pacemaker or defibrillator). The average of two seated blood pressure readings (Omron HEM-907 recorder,mmHg) were used. Region of residence (1978-80) was grouped into Scotland, North, Midlands and South of England.

**References**

1. Jefferis BJ, Sartini C, Lee IM, Choi M, Amuzu A, Gutierrez C, Casas JP, Ash S, Lennnon LT, Wannamethee SG, Whincup PH. Adherence to physical activity guidelines in older adults, using objectively measured physical activity in a population-based study. BMC Public Health 2014;14:382.

2. Choi L, Liu Z, Matthews CE, Buchowski MS. Physical Activity: Process Physical Activity Accelerometer Data (0.1-1). In. http://cran.r-project.org/; 2011.

**Table Appendix 1. Cross-sectional associations between physical activity intensity, sedentary time, and eGFR (<45 vs ≥45** **ml/min per 1.73m^2^) using CKD EPI cystatin, BIS2 and MDRD equations**

|  | **CKD EPI cys** | **n=1352** | **BIS2** | **n=1352** | **MDRD** | **n=1380** |
| --- | --- | --- | --- | --- | --- | --- |
|  | **OR** | **(95% CI)** | **OR** | **(95% CI)** | **OR** | **(95% CI)** |
|  |  |  |  |  |  |  |
| Total vertical counts (per 10,000/day) | **0.92** | **(0.89,0.95)** | **0.93** | **(0.90,0.96)** | **0.93** | **(0.90,0.97)** |
|  |  |  |  |  |  |  |
|  |  |  |  |  |  |  |
| steps (per 1000/day) | **0.76** | **(0.69,0.85)** | **0.78** | **(0.70,0.87)** | **0.83** | **(0.75,0.92)** |
|  |  |  |  |  |  |  |
|  |  |  |  |  |  |  |
| moderate/vigorous activity (10 mins/day) | **0.82** | **(0.75,0.91)** | **0.81** | **(0.74,0.90)** | **0.84** | **(0.76,0.93)** |
|  |  |  |  |  |  |  |
|  |  |  |  |  |  |  |
| light activity (30 mins/day) | **0.79** | **(0.71,0.87)** | **0.84** | **(0.76,0.94)** | **0.86** | **(0.78,0.96)** |
|  |  |  |  |  |  |  |
|  |  |  |  |  |  |  |
| sedentary behaviour (30 mins/day) | **1.25** | **(1.14,1.36)** | **1.19** | **(1.09,1.30)** | **1.16** | **(1.07,1.27)** |
|  |  |  |  |  |  |  |
|  |  |  |  |  |  |  |
| moderate/vigorous activity (10 mins/day) | 0.93 | (0.83,1.05) | **0.87** | **(0.76,0.98)** | 0.88 | (0.78,1.01) |
| sedentary behaviour (30 mins/day) | **1.19** | **(1.06,1.33)** | 1.09 | (0.97,1.22) | 1.07 | (0.95,1.21) |
|  |  |  |  |  |  |  |
|  |  |  |  |  |  |  |
| moderate/vigorous activity (10 mins/day) | **0.88** | **(0.80,0.97)** | **0.84** | **(0.76,0.94)** | **0.86** | **(0.77,0.96)** |
| light activity (30 mins/day) | **0.84** | **(0.75,0.94)** | 0.92 | (0.82,1.03) | 0.93 | (0.83,1.05) |
|  |  |  |  |  |  |  |

Bold type indicates significance at the 5% level.

eGFR estimated Glomerular filtration rate

CKD-EPI (Chronic Kidney Disease Epidemiology Collaboration) cystatin equation (gender term not required):

eGFR = 133 × (cystatin C/0.8)^−0.499^ × 0.996^age^ if cystatin C ≤ 0.8 mg/l and eGFR = 133 × (cystatin C /0.8)^−1.328^ × 0.996^age^ if cystatin C > 0.8 mg/l

BIS2 (Berlin Initiative Study) equation 2:

eGFR = 767 x cystatin C^-0.61^ x creatinine^-0.40^ x age^-0.57^

MDRD (Modification of Diet in Renal Disease) equation (race and gender terms not required):

eGFR = 175 x creatinine^-0.40^ x age^-0.203^

All models adjusted for daily accelerometer wear time, age and age squared, season of wear, region of residence, social class, living alone, tobacco, alcohol consumption, statin use, antihypertensive use, cardiovascular disease, diabetes, systolic blood pressure and total cholesterol
